# Effect of routine disinfection on bacterial composition and load of gym equipment surfaces in Qassim region, Saudi Arabia

**DOI:** 10.3389/fpubh.2026.1915360

**Published:** 2026-08-25

**Authors:** Noorah Saleh Al-Sowayan, Shouaa Abdulaziz Alrobaish, Ghadir Ghazi Al Suliman, Jana Fahad Alharbi, Norah Sulaiman Althiwaini, Wasayf Abdulrahman Almousa, Ghuyum Suliman Alrudhayman, Meznah Naif Alsalamah, Nariman Abdullah Alharbi

**Affiliations:** Department of Biology, College of Science, Qassim University, Buraydah, Saudi Arabia

**Keywords:** bacterial contamination, disinfectant efficacy, disinfection, fitness centers, *Micrococcus luteus*, *Staphylococcus*, VITEK 2, Wilcoxon test

## Abstract

**Background:**

Gym equipment surfaces represent high-contact environments that facilitate the transmission of bacterial pathogens between users. This study aimed to identify bacterial species contaminating gym equipment surfaces across six gyms in the Qassim Region, Saudi Arabia, and to evaluate the effectiveness of routine disinfection in reducing bacterial load and affecting bacterial composition.

**Methods:**

Twenty-one paired surface swab samples were collected before and after routine disinfection using sterile normal saline-moistened swabs from six gyms (A–F; one male, five female) on Sunday, April 5, 2026, between 7:00 and 8:00 p.m. during peak usage hours at the end of spring. Bacterial identification was performed using the VITEK 2 automated system, and colony counts were recorded on Blood Agar. The Wilcoxon Signed-Rank Test was applied for statistical analysis.

**Results:**

A total of 17 bacterial isolates were identified, distributed across three genera: *Micrococcus* (47.1%), *Staphylococcus* (47.1%), and *Kocuria* (5.9%). Post-disinfection colony counts decreased from 3,800 to 1,360 CFU, representing a 64.2% reduction, with a statistically significant difference (*Z* = 2.671, *p* = 0.008). Complete elimination was not achieved on all surfaces, and the treadmill in Gym B exhibited a post-disinfection increase from 180 to 370 CFU/100 cm², which may reflect rapid recontamination, normal sampling variation, or differences in the exact surface area sampled before and after disinfection. Notably, two of the six gyms (D and F) employed multi-purpose cleaning agents devoid of any biocidal active ingredient, suggesting that their procedures may provide cleaning activity rather than confirmed disinfection activity.

**Conclusion:**

These findings highlight the need for evidence-based disinfectants and standardized hygiene protocols in fitness facilities.

## Introduction

Gym equipment surfaces are among the most frequently touched surfaces in shared public environments, making them potential vehicles for bacterial transmission between users (1). As fitness center attendance has grown substantially worldwide, concerns regarding the microbial burden on equipment surfaces and the adequacy of disinfection protocols have intensified (1, 2). Despite the widespread adoption of routine cleaning procedures, their efficacy in reducing bacterial contamination remains insufficiently characterized (2).

Previous microbiological studies have documented diverse bacterial communities on gym equipment surfaces. Mukherjee et al. (1) reported a wide spectrum of bacterial communities on fitness center surfaces, predominantly of human skin origin. Bacterial loads on fitness equipment surfaces have been reported to range from 390 to 3,720 CFU/cm^2^, with *Escherichia coli* detected at 550–1,080 CFU/cm^2^ (3). Roberts et al. (2) reported a high prevalence of *Staphylococcus aureus* on gym equipment, positioning these surfaces as potential reservoirs for this pathogen—though Ryan et al. (4) found no MRSA or MSSA in 240 cultures from three community gyms, underscoring the variability across settings.

*Staphylococcus aureus* and its methicillin-resistant variant (MRSA) remain a primary public health concern in athletic environments (5). Zhang et al. (6) demonstrated that indoor gym surfaces harbor distinct microbial communities, with multi-drug-resistant strains including *Staphylococcus haemolyticus* identified on equipment surfaces. Beyond solid surfaces, microbial contamination also extends to the indoor air of fitness facilities, where genera such as Bacillus, Corynebacterium, Pseudomonas, and *Staphylococcus* dominate bioaerosols (7, 8). Goldhammer et al. (9) further observed that twice-daily disinfection of exercise equipment did not significantly reduce viral surface contamination, highlighting the inherent challenges of routine sanitation in high-use environments.

The detected bacteria in this study—including *Micrococcus luteus*, *Staphylococcus epidermidis*, and *Kocuria rosea*—are generally constituents of the normal human skin microbiome (1, 10). However, certain coagulase-negative staphylococci, such as *S. epidermidis*, *S. haemolyticus*, and *S. hominis*, are recognized opportunistic pathogens capable of causing serious infections in immunocompromised individuals or through direct contact with skin wounds sustained during exercise (2, 11).

This study addresses a gap in the regional literature, as no prior research has examined bacterial contamination in fitness centers in the Qassim Region. The findings are directly relevant to public health and align with Saudi Vision 2030 priorities in promoting quality of life and health awareness, as well as Sustainable Development Goals 3 (Good Health and Well-being) and 6 (Clean Water and Sanitation). Furthermore, the documentation of disinfectant types across all six gyms—revealing that two facilities employed non-biocidal cleaning agents—constitutes a novel contribution with direct practical implications for gym hygiene governance.

Beyond surface contamination, fitness facilities have been implicated in the transmission of skin and soft tissue infections, including those caused by *S. aureus* and its methicillin-resistant variant (MRSA). Fomite transmission via shared gym equipment is a recognized route of pathogen spread, particularly in high-density usage environments (2, 5). Of particular concern is the emergence of antimicrobial resistance (AMR) in isolates recovered from public gym surfaces; multidrug-resistant strains of *S. haemolyticus*, *Pseudomonas aeruginosa*, and *Klebsiella pneumoniae* have been documented in fitness center environments, raising significant implications for infection control and treatment efficacy (6, 12).

Human occupancy is considered the primary source of bacteria in indoor fitness environments (13), and environmental factors including elevated temperature and humidity—as observed during the spring sampling period—are known to promote microbial growth on surfaces.

### Research gap

Despite increasing global interest in the microbial ecology of fitness facilities, research addressing bacterial contamination in Saudi Arabian gyms—particularly in the Qassim Region—remains scarce. This study bridges three specific gaps:

- Absence of locally validated microbiological data on bacterial species colonizing fitness equipment in the Qassim Region.
- Lack of systematic evaluation of routine disinfection protocols in Saudi fitness facilities using culture-based and automated identification methods.
- No prior documentation of disinfectant types used in regional gyms or assessment of their biocidal adequacy.

This study addresses these gaps by providing field-based microbiological data from six gyms (one male, five female), collected during peak hours in the Qassim Region, with concurrent evaluation of disinfection efficacy and disinfectant characterization.

### Research question

This study seeks to answer the following primary research question:

Does routine disinfection as practiced in Qassim Region fitness centers significantly reduce bacterial load on equipment surfaces, and does the type of disinfectant used influence disinfection outcomes?

Two subsidiary questions are addressed:

1. What bacterial species predominate on the surfaces of fitness equipment in Qassim Region gyms?
2. Is there a difference in bacterial load reduction between gyms using true disinfectants and those using general-purpose cleaning agents?

### Research hypothesis

Based on the existing literature and the nature of the research problem, the following hypothesis is proposed:

#### Null hypothesis (H₀)

There is no statistically significant difference in bacterial load on fitness equipment surfaces in Qassim Region gyms before and after routine disinfection.

#### Alternative hypothesis (H₁)

There is a statistically significant difference in bacterial load on fitness equipment surfaces in Qassim Region gyms before and after routine disinfection, with a reduction in favor of post-disinfection measurements.

### Study objectives

This study aims to achieve the following objectives:

1. Identify bacterial species contaminating selected gym equipment surfaces in the Qassim Region.
2. Quantify bacterial load on these surfaces before and after routine disinfection.
3. Evaluate the effectiveness of routine disinfection protocols in reducing bacterial contamination.
4. Document and characterize the disinfectant products used across all participating gyms.
5. Compare bacterial load reduction between gyms using true disinfectants and those using general-purpose cleaning agents.
6. Provide evidence-based recommendations for improving hygiene practices in fitness facilities.

## Materials and methods

### Study design and sampling

A total of 21 surface swab samples were collected using sterile swab applicators (GlobalRoll, China) moistened with sterile normal saline from fitness equipment surfaces across six gyms (A–F) in the Qassim Region, Saudi Arabia (one male gym, five female gyms). The gyms had an estimated daily attendance ranging from approximately 50–800 users per day (as estimated by gym staff or gym members): Gym A (~70), Gym B (75–150), Gym C (400–500), Gym D (600–800), Gym E (100–120 during peak evening hours), and Gym F (approximately 50 users per day; lowest attendance among participating gyms). All gyms were non-mixed (five female-only gyms and one male-only gym). Sampling was conducted on Sunday, April 5, 2026, during peak usage hours at each facility (ranging from 7:00 p.m. to 8:00 p.m. depending on the gym), at the end of the spring season—a period associated with elevated ambient temperatures conducive to microbial proliferation. All surface samples were collected from a standardized 10 cm × 10 cm area.

The sample size of 21 surfaces was determined based on practical feasibility given the number of participating gyms (*n* = 6) and the constraint of single-visit sampling. Surfaces were selected using purposive sampling, targeting equipment with high user-contact frequency across all gyms. While this approach does not permit probabilistic inference, it maximizes ecological relevance for the study objectives. The culture method detects only viable, culturable bacteria and may underestimate total microbial diversity; non-culturable organisms and fungal or viral agents were beyond the scope of this study.

Sampled surfaces included: Pull up bar, Lat machine bar, Pectoral, Leg raise, Dumbbells, Locker door, Smith machine, Treadmill, Back extension, Fixed barbell rack, Cable machine handle, Rowing machine, Elliptical trainer, Bar, Chest fly, and Chair (locker room). Samples were collected in two phases:

- Phase 1: Immediately before disinfection.

### Disinfectants and surface sampling

- Phase 2: Immediately after disinfection, performed by the research team using the disinfectant product routinely available in each gym, ensuring standardized application across all surfaces. The disinfectant was allowed to remain on the surface for 10 min and until complete drying prior to collection of the post-disinfection sample. All participating gyms followed a uniform disinfection protocol: cleaning staff performed surface disinfection at opening time and at the beginning of the evening shift. Disinfectant products were made available at accessible locations throughout each gym for voluntary use by members prior to equipment use. No gym enforced mandatory post-use disinfection by members. Post-disinfection samples were collected immediately after the disinfected surfaces had completely air-dried. No additional fallow period was introduced, as the study aimed to evaluate routine disinfection practices under real-world gym conditions. No disinfectant neutralizing agent was used during post-disinfection swab collection.

Manufacturer-recommended contact times were not uniformly specified on product labels for all disinfectants used; where stated, contact times ranged from 30 s to 5 min. In this study, a standardized contact time of 10 min was applied to all surfaces to ensure comparability across gyms.

Each sterile swab was pre-moistened with 0.5 mL sterile normal saline and used to sample a defined surface area of 100 cm^2^. After sampling, the swab was transferred into a tube containing 10 mL sterile normal saline and mixed thoroughly. A 1 mL aliquot of the resulting suspension was inoculated onto the culture medium. Colony counts were multiplied by 10 to account for the total recovery volume and are expressed as CFU/100 cm^2^.

The disinfectant products used in each gym were documented and characterized by active ingredient and chemical class, as detailed in Table 1. All six gyms employed commercially available products; however, two gyms (D and F) used multi-purpose cleaning agents lacking any recognized biocidal active ingredient.

**Table 1:** Disinfectant products used across the six participating gyms.

Gym	Product	Active ingredient	Concentration	Chemical class
A	Dafa hand sanitizer	Ethanol	70%	Ethyl alcohol
B	SPI-gel	Ethanol	70%	Ethyl alcohol
C	STERI-7 extra	Benzalkonium Cl + didecyl dimethyl ammonium Cl	Not specified	Quaternary ammonium
D	Sutter diamond*	Plant-based surfactants only	Not specified	Cleaning agent—no confirmed biocidal active ingredient
E	Dettol	Chloroxylenol	4.8% w/v	Phenolic
F	3DX germs killer*	Glycol butyl ether + KOH + surfactants	Not specified	Cleaning agent—no confirmed biocidal active ingredient

KOH, potassium hydroxide; QAC, quaternary ammonium compound. ^*^Products with no biocidal active ingredient.

### Microbiological analysis

Swab samples were plated onto Blood Agar using a quantitative culture technique and incubated aerobically at 37 °C for 24–48 h. Colony counts were recorded as colony-forming units (CFU) recovered from a standardized 100 cm^2^ surface area and are reported as CFU/100 cm^2^. Blood Agar was selected as a general-purpose enriched medium suitable for recovering a broad range of culturable aerobic bacteria from environmental surface samples. However, bacteria requiring selective media, anaerobic conditions, or specialized growth requirements may not have been recovered.

Representative colonies exhibiting distinct morphological characteristics (e.g., differences in colony size, shape, color, texture, and appearance on Blood Agar) were selected for identification using the VITEK 2 system. Colonies with similar morphology from the same sample were not repeatedly analyzed.

Representative Gram-positive cocci were identified using VITEK 2 GP identification cards (bioMérieux, France). No other VITEK 2 card types (GN, BCL, or YST) were used, as the investigation focused on clinically significant Gram-positive cocci. Confidence levels for each identification were recorded and reported.

The VITEK 2 system (bioMérieux, Marcy-l’Étoile, France; software version 9.02) was used for automated biochemical identification. Quality control was performed using reference strains *S. aureus* ATCC 25923 and *S. epidermidis* ATCC 12228 prior to sample analysis. The system was calibrated according to manufacturer specifications before each analytical run.

### Statistical analysis

The Wilcoxon Signed-Rank Test was applied to compare pre- and post-disinfection colony counts, given the non-parametric distribution of the data. Statistical significance was defined at *α* ≤ 0.05.

In addition to inferential statistics, descriptive statistics were computed for pre- and post-disinfection colony counts, including median, interquartile range (IQR), minimum, and maximum values, to characterize the distribution of bacterial load across samples. These are presented in Table 2.

**Table 2:** Descriptive statistics for pre- and post-disinfection bacterial load (CFU/100 cm^2^).

Variable	Mean	Median	IQR	Min	Max
Pre-disinfection (CFU)	181.0	30	140	0	2,330
Post-disinfection (CFU)	65.2	0	10	0	870

Ethical Considerations: This study did not involve any direct human subjects or personal data collection. All samples were obtained exclusively from equipment surfaces without interaction with gym users or access to personal information. Formal ethical approval was therefore not required, as the study involved environmental surface sampling only and did not include human participants or personal data collection. Permission to conduct sampling was obtained from the management of all participating fitness centers prior to sample collection. All microbiological procedures were conducted in accordance with standard laboratory biosafety practices in the Department of Biology, College of Science, Qassim University. Bacterial cultures and contaminated materials were handled, stored, and disposed of following institutional laboratory safety procedures.

## Results

### Bacterial load: pre- and post-disinfection comparison

Post-disinfection bacterial load decreased significantly across all 21 samples, from a total of 3,800 CFU/100 cm^2^–1,360 CFU/100 cm^2^, representing a 64.2% reduction. The Wilcoxon Signed-Rank Test confirmed this reduction was statistically significant (*Z* = 2.671, *p* = 0.008). Table 3 presents detailed results for each sample.

**Table 3:** Comparison of bacterial load (CFU/100 cm^2^) before and after disinfection for all samples (*n* = 21).

Sample ID	Surface type	Pre-disinfection (CFU/100 cm^2^)	Post-disinfection (CFU/100 cm^2^)	Change (%)
1-A	Chest fly	0	0	—
2-A	Dumbbells	50	0	↓ 100%
3-A	Chair (locker room)	0	0	—
4-B	Dumbbells	300	10	↓ 96.7%
5-B	Smith machine (bench)	2,330	870	↓ 62.7%
6-B	Treadmill	180	370	↑ +105.6%
7-C	Leg raise	20	0	↓ 100%
8-C	Dumbbells	150	10	↓ 93.3%
9-C	Locker door	10	0	↓ 100%
10-D	Pull up bar	0	0	—
11-D	Lat machine bar	250	0	↓ 100%
12-D	Pectoral	30	10	↓ 66.7%
13-E	Back extension	220	0	↓ 100%
14-E	Fixed barbell rack	30	0	↓ 100%
15-E	Cable machine handle	10	10	No change
16-E	Dumbbells	20	0	↓ 100%
17-E	Lat pulldown bar	10	10	No change
18-E	Dumbbells	110	10	↓ 90.9%
19-F	Rowing machine	70	50	↓ 28.6%
20-F	Elliptical trainer	10	10	No change
21-F	Bar	0	0	—
Total		3,800	1,360	↓ 64.2%

CFU, colony forming unit; ↓, decrease (green); ↑, increase (red). —, no growth detected in either phase.

### Wilcoxon signed-rank test results

Table 4 summarizes the statistical output of the Wilcoxon test:

**Table 4:** Wilcoxon signed-rank test results for pre- vs. post-disinfection bacterial load.

Variable	*N*	Mean	Std Dev	Ranks	Mean rank	*p*-value
Pre-disinfection	21	181.0	501.2	Negative ranks: 13	7.31	**0.008**
Post-disinfection	21	65.2	201.1	Positive ranks: 1|Ties: 7	10.00	

The Wilcoxon W statistic (sum of positive ranks) = 91. This value represents the sum of ranks for the single positive rank (post > pre) observed in the dataset. The *Z* approximation (*Z* = 2.671) and exact *p*-value (*p* = 0.008) are reported as the primary test statistics.

^*^Statistically significant at *α* ≤ 0.05. *Z* = Wilcoxon test statistic. *p* = significance level. Difference in favor of higher pre-disinfection mean.

Thirteen negative ranks (post < pre) were recorded, compared to one positive rank (post > pre) and seven ties (no change), confirming a consistent reduction in bacterial load following disinfection.

### Bacterial composition: species distribution

A total of 17 representative bacterial isolates exhibiting distinct colony morphologies were selected for identification using the VITEK 2 system. The identified isolates represent selected culturable bacteria recovered from sampled surfaces and may not fully reflect the complete bacterial composition of those surfaces. Table 5 presents the distribution of identified species.

**Table 5:** Distribution of bacterial species identified by VITEK 2 (*n* = 17).

Species	Count	Percentage (%)
*Micrococcus luteus*	8	47.1%
*Staphylococcus epidermidis*	2	11.8%
*Staphylococcus haemolyticus*	2	11.8%
*Staphylococcus warneri*	2	11.8%
*Staphylococcus hominis ssp. hominis*	2	11.8%
*Kocuria rosea*	1	5.9%
Total	17	100%

Source: VITEK 2 automated identification system | Percentages calculated from 17 total isolates.

### Bacterial composition: genus distribution

The 17 isolates belonged to three genera, as presented in Table 6.

**Table 6:** Distribution of bacterial genera identified by VITEK 2.

Genus	Count	Percentage (%)
*Micrococcus*	8	47.1%
*Staphylococcus*	8	47.1%
*Kocuria*	1	5.9%
Total	17	100%

Source: VITEK 2 automated identification system.

### VITEK 2 identification confidence levels

Confidence levels for species identification by VITEK 2 are presented in Table 7:

**Table 7:** VITEK 2 identification confidence levels for all bacterial isolates.

Confidence level	Count	Percentage (%)
Excellent	13	76.47%
Very good	1	5.88%
Good	1	5.88%
Low discrimination	2	11.76%
Total	17	100%

Source: VITEK 2 automated identification system.

### Key observations

- Seven surfaces achieved complete bacterial elimination following disinfection (positive pre-disinfection count reduced to zero): Dumbbells (2-A), Leg raise (7-C), Locker door (9-C), Lat machine bar (11-D), Back extension (13-E), Fixed barbell rack (14-E), and Dumbbells (16-E).
- Seven surfaces retained detectable contamination following disinfection: Dumbbells (4-B), Smith machine (5-B), Treadmill (6-B), Dumbbells (8-C), Pectoral (12-D), Dumbbells (18-E), and Rowing machine (19-F).
- Three surfaces showed no change in bacterial load: Lat pulldown bar (17-E), Cable machine handle (15-E), and Elliptical trainer (20-F).
- Four surfaces showed no bacterial growth in either phase (pre- and post-disinfection both zero): Chest fly (1-A), Chair, locker room (3-A), Pull up bar (10-D), and Bar (21-F).
- The Treadmill (6-B) exhibited a post-disinfection increase from 180 to 370 CFU/100 cm^2^ (+105.6%), possibly indicating recontamination.
- The Smith machine (5-B) recorded the highest pre-disinfection contamination at 2,330 CFU/100 cm^2^, reduced to 870 CFU/100 cm^2^ post-disinfection.
- VITEK 2 identification confidence was rated Excellent in 76.47% of isolates, supporting the reliability of species-level results. Photographic documentation of all Blood Agar plates before and after disinfection is provided in Appendix Figures 1–4.
- Disinfectant documentation revealed that Gyms D and F employed multi-purpose cleaning agents lacking biocidal active ingredients, while Gyms A, B, C, and E used recognized chemical disinfectants. This finding is summarized in Table 1 and represents a key public health observation of this study.

### Descriptive comparison: true disinfectants vs. cleaning agents

Based on disinfectant classification, a descriptive comparison was conducted between gyms using true biocidal disinfectants (A, B, C, E) and those using general-purpose cleaning agents (D, F), as shown in Table 8:

**Table 8:** Descriptive comparison of bacterial load reduction (CFU/100 cm^2^) between disinfectant groups.

Group	PRE total (CFU/100 cm^2^)	POST total (CFU/100 cm^2^)	Reduction (CFU/100 cm^2^)	Reduction (%)
True disinfectants (A, B, C, E)	3,440	1,290	2,150	62.5%
Detergents only (D, F)	360	70	290	80.6%*
Overall total	3,800	1,360	2,440	64.2%

^*^The higher percentage reduction in the detergent group is attributable to a substantially lower baseline bacterial load (360 vs. 3,440 CFU/100 cm^2^), not greater disinfectant efficacy. The absolute reduction in the true disinfectant group was markedly higher (2,150 vs. 290 CFU/100 cm^2^).

Despite the seemingly higher percentage reduction in Gyms D and F (80.6% vs. 62.5%), their baseline contamination was substantially lower (360 vs. 3,440 CFU), suggesting lower user density or usage frequency rather than superior disinfectant performance.

Descriptive comparison suggested differences in percentage reduction between disinfectant groups; however, no formal statistical comparison was performed due to the limited and unequal sample sizes between groups (*n* = 4 vs. *n* = 2). These observations should therefore be interpreted with caution and are intended to generate hypotheses for future controlled studies.

## Discussion

The bacterial isolates recovered from fitness equipment surfaces in the Qassim Region were predominantly members of the normal human skin microbiome — including *M. luteus*, *S. epidermidis*, and *K. rosea* (1, 10)—consistent with the established role of human occupancy as the primary source of bacteria in indoor fitness environments (13). These species are generally non-pathogenic in healthy individuals; however, coagulase-negative staphylococci such as *S. haemolyticus* and *S. hominis* are recognized opportunistic pathogens capable of causing serious infections in immunocompromised individuals or through direct contact with cutaneous wounds incurred during exercise (2, 11).

The Wilcoxon Signed-Rank Test confirmed a statistically significant reduction in total bacterial load following routine disinfection (*Z* = 2.671, *p* = 0.008; 64.2% reduction), consistent with previous findings indicating that routine disinfection reduces—but does not eliminate—surface contamination in fitness settings (3, 9, 14). Toh et al. (14) similarly observed that alcohol-based wipe disinfection in a rehabilitation gym environment significantly reduced microbial transmission, supporting the use of ethanol-based products (as in Gyms A and B) as a frontline disinfection strategy.

Similar concerns regarding hygiene practices and microbial contamination in fitness facilities have also been reported by Batista et al. (15), who highlighted the importance of routine monitoring and hygiene assessment in gym environments.

### Biofilm formation and disinfectant resistance

A key mechanism explaining the persistence of bacterial contamination on gym surfaces—despite routine disinfection—is the capacity of certain species to form biofilms. *Staphylococcus epidermidis*, identified at a frequency of 11.8% in this study, is well recognized for its strong biofilm-forming ability on both metallic and plastic surfaces (11). Biofilms are structured communities of bacteria embedded in a self-produced extracellular polymeric matrix, which acts as a physical and biochemical barrier that substantially reduces the penetration of disinfectants to the underlying cells, potentially requiring up to 1,000-fold higher biocide concentrations to achieve comparable killing efficacy against planktonic cells (15). *Staphylococcus haemolyticus*—also identified in this study—similarly demonstrates biofilm-forming capacity and is further characterized by multidrug resistance, including resistance to methicillin, aminoglycosides, and fluoroquinolones (11). The persistence of contamination on textured or mechanically complex surfaces (e.g., the Smith machine bench, 5-B: 2,330 → 870 CFU) is consistent with biofilm-related resistance, as surface irregularities provide protected niches for microbial adhesion and biofilm maturation that are difficult to penetrate with standard wiping disinfection protocols.

A critical finding of this study is the identification of two gyms (D and F) employing general-purpose cleaning agents—Sutter Diamond and 3DX, respectively—that contain no recognized biocidal active ingredient. This suggests that their procedures may provide cleaning activity rather than confirmed disinfection activity. The use of 70% ethanol (Gyms A and B), Quaternary Ammonium Compounds (Gym C), and Chloroxylenol (Gym E/Dettol) represents evidence-based disinfection. Liao et al. (16) confirmed the bactericidal efficacy of 70% ethanol against *Staphylococcus* species, supporting its continued use in high-contact surface disinfection.

The apparent paradox of a higher percentage reduction in Gyms D and F (80.6%) compared to Gyms A, B, C, and E (62.5%) is explained by substantially lower baseline bacterial load in the former group (360 vs. 3,440 CFU), likely reflecting lower user density or equipment usage frequency rather than any disinfectant efficacy.

The post-disinfection increase observed on the Treadmill in Gym B (6-B; 180 → 370 CFU, +105.6%)—despite the use of 70% ethanol—may suggest rapid recontamination, possibly attributable to immediate resumption of equipment use following cleaning or cross-contamination from cleaning materials. This observation is consistent with the findings of Hospodsky et al. (13), who identified human occupancy as the dominant source of indoor airborne bacteria in fitness environments.

The post-disinfection increase on the Treadmill (6-B) warrants particular attention. Several mechanisms may account for this observation: (1) aerosol deposition of bacteria from adjacent users during or immediately after disinfection; (2) recontamination via contaminated cleaning cloths or applicators; (3) inadequate surface drying time prior to reuse; and (4) high user traffic volume in Gym B (75–150 users/day), which may result in near-immediate recontamination following cleaning. This finding underscores that routine cleaning ≠ disinfection, particularly in high-traffic environments where surface recontamination can occur within minutes of cleaning completion. Furthermore, *S. haemolyticus*—one of the species identified in this study—is commonly associated with multidrug resistance, including resistance to methicillin, aminoglycosides, and fluoroquinolones, even without formal resistance testing in the current study (11). While antimicrobial susceptibility was beyond the scope of this investigation, the presence of this species on high-contact surfaces represents a potential public health concern that merits follow-up investigation.

Surface complexity appears to mediate disinfection outcomes: smooth, simple surfaces such as the Lat machine bar and locker door achieved complete bacterial elimination, whereas structurally complex or high-contact equipment (e.g., Smith machine) retained contamination—concordant with data reported by Szulc et al. (7).

Regarding VITEK 2 identification reliability, an Excellent confidence rating was achieved for 76.47% of isolates, validating the taxonomic accuracy of the results. The equal predominance of *Micrococcus* and *Staphylococcus* genera (47.1% each) aligns with the findings of Mukherjee et al. (1), who similarly observed the dominance of these skin-associated genera on fitness center surfaces.

Viegas et al. (8) reported widespread bacterial and fungal contamination in Portuguese fitness centers, including Gram-negative bacteria associated with antimicrobial resistance—reinforcing the broader public health relevance of gym surface hygiene across geographic contexts.

The sampling time (7:00–8:00 p.m. during peak evening hours at the end of spring) provides ecological relevance; elevated ambient temperatures typical of the spring season in Qassim may have promoted microbial proliferation on equipment surfaces, and the peak-hour context ensures that results reflect real-world conditions of maximum user exposure.

These findings align with Saudi Vision 2030 priorities of promoting public health and quality of life in sports and fitness facilities, and support Sustainable Development Goals 3 (Good Health and Well-being) and 6 (Sanitation and Hygiene) (17).

## Conclusion

This study confirmed the presence of bacterial contamination on fitness equipment surfaces across six gyms in the Qassim Region, Saudi Arabia, predominantly involving *Micrococcus*, *Staphylococcus*, and *Kocuria* genera. Routine disinfection significantly reduced total bacterial load by 64.2% (3,800 → 1,360 CFU; *p* = 0.008), yet did not completely eliminate bacterial contamination on all surfaces. A post-disinfection increase was observed on the Treadmill in Gym B (6-B), which may reflect rapid recontamination, normal sampling variation, or differences in the exact surface area sampled before and after disinfection.

A key finding of public health significance is that two of the six participating gyms employed multi-purpose cleaning agents lacking biocidal properties, constituting surface cleaning rather than true disinfection. This represents a previously undocumented gap in gym sanitation practices in the region.

These results collectively underscore the need for systematic review of disinfection protocols in fitness facilities, informed selection of evidence-based disinfectants, and enhanced staff training in sanitation practices. The study contributes to the regional evidence base supporting Saudi Vision 2030 and the relevant Sustainable Development Goals.

### Study limitations

The following limitations should be considered when interpreting the findings of this study:

- Samples were collected on a single day from a limited number of fitness centers within one geographic region, which may restrict the generalizability of the findings across different seasons, locations, and usage conditions.
- The investigated surfaces differed among gyms according to equipment availability, limiting direct comparisons between facilities.
- Fungal contamination of gym surfaces was not assessed. Given the potential clinical relevance of mold and dermatophyte contamination in fitness environments—particularly in locker room areas—this represents a gap that future studies should address.
- Viral surface contamination was not evaluated. Fitness centers are recognized settings for fomite-mediated transmission of respiratory and enteric viruses; the microbial safety assessment in this study was therefore limited to culturable bacteria.
- Post-disinfection sampling was performed at a single time point (ten minutes after disinfectant application). The temporal dynamics of surface recontamination over longer intervals (e.g., 30 min, 1 h) were not evaluated, which limits conclusions about the durability of disinfection effectiveness.
- The study did not include a standardized fallow period following disinfection, which may have influenced post-disinfection bacterial recovery.
- User compliance with voluntary hygiene practices—including self-disinfection of equipment before use—was not monitored or quantified. Differential compliance across gyms may have influenced post-disinfection bacterial counts independently of the disinfectant product used.
- Environmental parameters, including indoor temperature and relative humidity, were not measured and may have influenced bacterial counts.
- The study relied on culture-based methods and did not include molecular techniques, which may have provided a more comprehensive characterization of the microbial community.
- Only representative bacterial colonies exhibiting distinct morphological characteristics were selected for identification using the VITEK 2 system. Therefore, the identified isolates may not fully represent the complete bacterial composition present on all sampled surfaces.
- The relatively small sample size (21 swabs collected from six gyms) may limit broader generalization of the results to all fitness centers in the region.

### Recommendations

Based on the findings of this study, the following recommendations are proposed:

1. Gyms D and F are encouraged to consider replacing their multi-purpose cleaning agents with evidence-based disinfectants containing recognized biocidal active ingredients (e.g., ethanol ≥70%, quaternary ammonium compounds, or chloroxylenol).
2. Special attention should be directed to structurally complex equipment such as the Smith machine (5-B), which exhibited the highest contamination levels.
3. A defined post-disinfection safety interval should be established before equipment is returned to use, to prevent the recontamination pattern observed on the Treadmill (6-B).
4. Disinfection frequency should be increased, particularly during peak usage hours.
5. Gym staff should receive standardized training in evidence-based disinfection techniques.
6. Gym users should be encouraged to practice personal hygiene, including the use of personal towels and hand sanitizers.
7. Future studies should include a larger sample size, multiple sampling time points across seasons, and comparative trials of specific disinfectant formulations.

Routine microbiological surveillance of gym equipment surfaces should be incorporated into facility hygiene quality assurance programs, with standardized sampling protocols and minimum acceptable CFU thresholds defined by public health authorities.

## Data Availability

The original contributions presented in the study are included in the article/Supplementary material, further inquiries can be directed to the corresponding author.
