# Supplementary material for: Effect of routine disinfection on bacterial composition and load of gym equipment surfaces in Qassim region, Saudi Arabia
Source: Front Public Health. 2026 Aug 25;14:1915360. doi: 10.3389/fpubh.2026.1915360 (PMC13550117; doi:10.3389/fpubh.2026.1915360)
Supplement: Supplementary file 1 [file Data_Sheet_1.docx]

**Appendix: Blood Agar Plate Photographs — Pre- and Post-Disinfection (Figures 1–4)**

Figures 1–4 present Blood Agar culture plates for four representative samples selected to illustrate the key findings of this study: the surface with highest contamination (Smith machine), the surface that exhibited a post-disinfection increase (Treadmill), a representative sample (Dumbbells), and a surface achieving complete bacterial elimination (Lat machine bar). Photographs of all 21 samples are provided in the Supplementary Appendix.

| **Figure (1)**  **5-B**  Smith machine (bench)  Gym B |   **Pre-Disinfection** |   **Post-Disinfection** |
| --- | --- | --- |

*Highest contamination (2,330 CFU/100 cm² pre-disinfection; 870 CFU/100 cm² post)*

| **Figure (2)**  **6-B**  Treadmill  Gym B |   **Pre-Disinfection** |   **Post-Disinfection** |
| --- | --- | --- |

*Post-disinfection increase (180 → 370 CFU/100 cm²); may indicate recontamination*

| **Figure (3)**  **8-C**  Dumbbells  Gym C |   **Pre-Disinfection** |   **Post-Disinfection** |
| --- | --- | --- |

*Representative sample (15 → 1 CFU; 93.3% reduction)*

| **Figure (4)**  **11-D**  Lat machine bar  Gym D |   **Pre-Disinfection** |   **Post-Disinfection** |
| --- | --- | --- |

*Complete elimination (250 → 0 CFU/100 cm²)*

**Supplementary Data Note**

Blood Agar plate photographs for all 21 samples (pre- and post-disinfection) are available in the Supplementary Appendix accompanying this manuscript.
